# The incidence of falls after first and second eye cataract surgery: a longitudinal cohort study

**DOI:** 10.5694/mja2.51611

**Published:** 2022-06-15

**Authors:** Lisa Keay, Kam Chun Ho, Kris Rogers, Peter McCluskey, Andrew JR White, Nigel Morlet, Jonathon Q Ng, Ecosse Lamoureux, Konrad Pesudovs, Fiona J Stapleton, Soufiane Boufous, Jessie Huang‐Lung, Anna Palagyi

**Affiliations:** ^1^ The University of New South Wales Sydney NSW; ^2^ The George Institute for Global Health Sydney NSW; ^3^ The University of Canberra Canberra ACT; ^4^ Save Sight Institute The University of Sydney Sydney NSW; ^5^ Westmead Institute for Medical Research Sydney NSW; ^6^ The University of Western Australia Perth WA; ^7^ Duke‐NUS Medical School Singapore Singapore; ^8^ Transport and Road Safety (TARS) Research Centre University of New South Wales Sydney NSW

**Keywords:** Cataract, Hospitals, Falls, Accident prevention, Health planning

## Abstract

**Objective:**

To compare fall incidence, and visual acuity and refractive status, before surgery and after first and second eye cataract surgery.

**Design, setting:**

Prospective observational study in eight tertiary referral ophthalmology clinics in public hospitals in Sydney, Melbourne, and Perth.

**Participants:**

People aged 65 years or more referred for bilateral age‐related cataract surgery during 2013–16, followed for maximum of 24 months after study entry or until six months after second eye surgery, whichever was shorter.

**Main outcome measures:**

Primary outcome: age‐ and sex‐adjusted incidence of falls. Secondary outcomes: visual acuity and refractive error.

**Results:**

The mean age of the 409 included participants was 75.4 years (SD, 5.4 years); 220 were women (54%). Age‐ and sex‐adjusted fall incidence prior to surgery was 1.17 (95% CI, 0.95–1.43) per year, 0.81 (95% CI, 0.63–1.04) per year after first eye surgery, and 0.41 (95% CI, 0.29–0.57) per year after second eye surgery. For the 118 participants who underwent second eye surgery and participated in all follow‐up visits, age‐ and sex‐adjusted incidence before (0.80 [95% CI, 0.55–1.15] falls per year) and after first eye surgery (0.81 [95% CI, 0.57–1.15] falls per year) was similar, but was lower after second eye surgery (0.32 [95% CI 0.21–0.50] falls per year). Mean habitual binocular visual acuity (logMAR) was 0.32 (SD, 0.21) before surgery, 0.15 (SD, 0.17) after first eye surgery, and 0.07 (SD, 0.15) after second eye surgery.

**Conclusions:**

First eye surgery substantially improves vision in older people with cataract, but second eye surgery is required to minimise fall incidence. Timely cataract surgery for both eyes not only optimises vision in older people with cataract, but also reduces their risk of injury from falls.

**The known**: First eye cataract surgery reduces the risk of falls in older people, but the effect of second eye cataract surgery is less clear.

**The new**: The age‐ and sex‐adjusted incidence of falls among older people referred for cataract surgery was 31% lower after first eye surgery, and a further 50% lower after second eye surgery, restoring binocular vision.

**The implications**: Timely and equitable access to cataract surgery can prevent fall‐related injuries and support healthy ageing.

Cataract is a leading cause of vision impairment in Australia, despite the effectiveness^1^ and availability of cataract surgery.^2^ Cataract‐related vision impairment (logMAR, 0.3 [Snellen, 6/12] or worse) affects 2.7% (95% confidence interval [CI], 2.0–3.5%) of non‐Indigenous Australians aged 50 years or more.^3^ Major disparities in access to cataract surgery have been reported in Australia and other high income countries.^4^

A meta‐analysis of six quasi‐experimental studies^5^ found that cataract surgery reduces the frequency of falls by older people by 32% (relative risk [RR], 0.68; 95% CI, 0.48–0.96), but the authors did not differentiate between first and second eye surgery. The only randomised controlled trial (RCT) of expedited cataract surgery (undertaken in the United Kingdom) found that first eye cataract surgery reduced the fall rate by 34% (RR, 0.66; 95% CI, 0.45–0.96);^6^ the authors’ subsequent second eye surgery RCT did not find a statistically significant reduction (RR, 0.68; 95% CI, 0.39–1.19).^7^ Data linkage studies have yielded mixed results with respect to the effects of first and second eye surgery on the incidence of falls leading to hospitalisation, and not all potentially confounding factors were taken into account.^8^, ^9^

Our recent cohort study of fall risk, Falls in Older people with Cataract: a longitudinal evaluation of impact and risk (FOCUS)^10^ distinguished between the preventive effects of first eye surgery (incidence rate ratio [IRR], 0.67; 95% CI, 0.49–0.92) and the influence of other factors, including poor pre‐surgery vision in the dominant eye (IRR, 2.20; 95% CI, 1.02–4.74) and large post‐surgery changes in spherical equivalent spectacle lens correction (0.75 dioptre or more: IRR, 2.17; 95% CI, 1.23–3.85).^11^ In this article, we compare fall risk, and visual acuity and refractive error as secondary outcomes, before surgery and after first and second eye cataract surgery.

## Methods

The FOCUS study was a prospective observational study of the influence of cataract surgery on falls, vision, and refractive status.^10^ People aged 65 years or more referred for bilateral age‐related cataract surgery were identified in surgery waiting lists (1 October 2013 – 31 July 2016) at eight Australian public hospitals: the Sydney Eye, Westmead, Bankstown, and Royal North Shore Hospitals (Sydney), the Royal Victorian Eye and Ear Hospital (Melbourne), and the Fremantle, Royal Perth, and Sir Charles Gairdner Hospitals (Perth). Potential participants were invited by letter and contacted by phone one week later to ascertain their interest and to screen them for eligibility. In pre‐recruitment assessments, we excluded people who made more than two mistakes on the Short Portable Mental Status Questionnaire,^12^ reported dementia, Parkinson disease or stroke, were unable to walk (aided or unaided), resided in residential aged care facilities, had other major ocular conditions (eg, glaucoma, diabetic retinopathy, age‐related macular degeneration), planned combined surgery (eg, trabeculectomy and cataract surgery), or could not complete study assessments in English. Participants were followed for six months after second eye surgery or for two years after recruitment, whichever period was shorter. Data collection ended on 30 June 2018.

### Assessments

Trained research assistants undertook baseline and follow‐up assessments using standardised protocols. Follow‐up assessments were performed about three months after surgery; they were not undertaken after first eye surgery if the scheduled time to second eye surgery was less than three months. Date of birth, sex, height, and weight data were collected at baseline. A medical history was taken and physical and vision‐related assessments were performed at each study visit, including self‐reported medication use and physician‐diagnosed medical conditions (combined as a summary measure, the Functional Comorbidities Index, FCI^13^), physical activity (Incidental and Planned Exercise Questionnaire^14^), and physical function (Short Physical Performance Battery, SPPB^15^). Component scores for a timed 4 m walk (m/s), standing balance for ten seconds in six stances (range, 0–60 s), and five sit‐to‐stand repetitions (stands per second) were combined as the SPPB score (0–12; 0 = poorest, 12 = best physical performance).

The type of spectacles usually worn by the participant was recorded, and monocular and binocular high contrast visual acuity at 3 m were measured with the Early Treatment Diabetic Retinopathy Study chart,^16^ with habitual distance correction in standard room illumination (minimum, 480 lux). Monocular and binocular contrast sensitivity were measured with habitual near correction using the Mars letter contrast sensitivity test^17^ at 50 cm. Refractive error was assessed by auto‐refraction (using the vector length equation: |P| = √[(*S* + *C*/2)^2^ + (–*C*/2cos2θ)^2^ + (–*C*/2sin2θ)^2^]; *S* = spherical power, *C* = cylindrical power, θ = axis for sphero‐cylindrical power specified in negative cylinder form).^18^ Stereopsis was evaluated with the Titmus Fly stereo test;^19^ we report the proportion of participants with at least moderate stereopsis (140 seconds of arc) on the Wirt circles.

### Falls data

The primary outcome was the incidence of falls, defined as “an unexpected event in which the participant comes to rest on the ground, floor, or lower level”.^20^ Falls data were collected prospectively in monthly self‐report calendars, from study entry to 24 months after entry or six months after second eye surgery, whichever was earlier. Interviewers contacted participants by phone if monthly falls calendars were not returned to obtain information about the circumstances of any falls. The interviewers confirmed surgery dates for participants, but were unaware of our study hypothesis.

### Sample size

The FOCUS study recruitment target (652 participants; 90% power to detect a difference in proportions of 0.05 in McNemar test power analysis, undertaken in PASS 11 [NCSS]), was not achieved.^10^ The target was based on fall prevalence in an earlier study of people with cataracts,^6^ a six‐month observation period, and the proportions of discordant pairs (falls before and after surgery) in a pilot study. The primary outcome (falls before^21^ and after first eye surgery^11^) and the secondary outcomes (depressive symptoms,^22^ physical activity, and fear of falling^23^) were assessed using the data for the first 329 participants. As the decline in number of falls was greater than anticipated, a sample size of 329 participants provided 80% power to detect a 32% reduction in fall incidence,^11^ assuming a mean pre‐surgery exposure time of 0.88 years and overdispersion parameter of 1.5. Phase 2 recruitment, limited by resource constraints to assessing the primary outcomes (falls and vision; Supporting Information), ended in July 2016; all collected data were available for our analysis.

### Statistical analysis

Three time periods were defined: enrolment to first eye surgery (pre‐surgery), the period between first and second eye surgery, and the period after second eye surgery. If assessments were not completed, data were treated as missing and not imputed. Age, sex, health, and visual and physical function data are summarised as descriptive statistics. We report statistically significant differences (as mean differences with 95% CIs) in baseline characteristics between participants who did not undergo surgery, underwent first eye surgery only, or underwent first and second eye surgery; and in vision characteristics between time periods. We estimated the age‐ and sex‐adjusted incidence of falls and incident rate ratios for the three time periods in a negative binomial regression model, confirmed as appropriate by checking the dispersion parameter; we used number of observation days as the offset to adjust for the different lengths of the three periods. Informed by our previous work,^11^, ^21^ we chose a minimally adjusted model that took collinearity of vision‐related variables and surgical status into account. An exchangeable correlation structure by participant accounted for repeated measures in individuals. All analyses used general linear models (in SAS Enterprise Guide 7.1) to adjust for inherent correlation of repeated measures (α = 0.05). Our study report complies with the STROBE guideline.^24^

### Ethics approval

Ethics approval was granted by the NSW Population and Health Services Research Ethics Committee (HREC/13/CIPHS/25), and the human research ethics committees of Curtin University (HR 123/2013) and the Royal Victorian Eye and Ear Hospital (13/1124H). All participants provided written, informed consent.

## Results

A total of 416 of 1664 invited people agreed to participate in our study (25%). Six were subsequently excluded because prospective falls data were not available, and one because the recorded dates of surgery in their hospital records indicated that they did not have bilateral age‐related cataract at baseline. The mean age of the 409 included participants was 75.4 years (standard deviation [SD], 5.4 years); 220 were women (54%). Sixty‐three participants (15%) did not undergo cataract surgery during the study period. Median time from enrolment to first eye surgery for 346 participants was 176 days (interquartile range [IQR], 89–321 days). A total of 188 participants (46%) progressed to second eye cataract surgery; the median time between first and second eye surgery was 265 days (IQR, 119–493 days), and after second eye surgery was 211 days (IQR, 189–225 days) (Box 1).

Box 1 **Flow of the 416 enrolled participants through the study**

**Figure:**
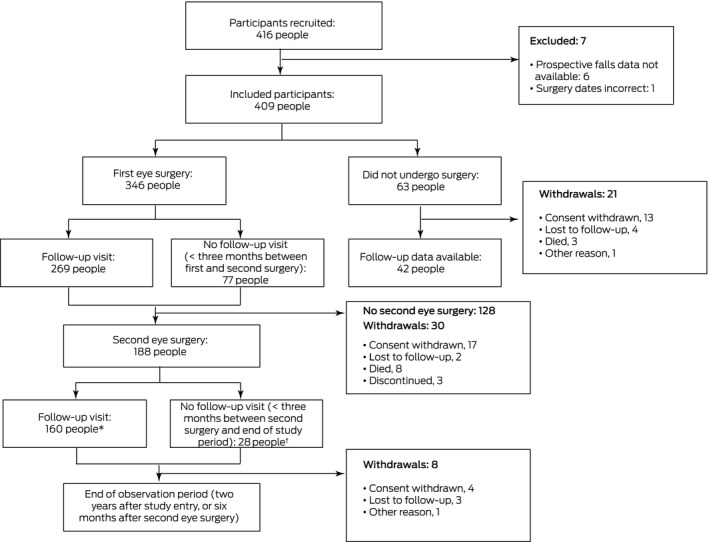
^*^ Includes 42 participants who had follow‐up visits after second eye surgery, but not after the first operation. ^†^ Includes ten participants who had second eye surgery within one month of first eye surgery.

### Visual performance

At the time of enrolment, habitual vision was poorer for participants who later progressed to second eye surgery than for those who had first eye surgery only (mean difference [logMAR], 0.09; 95% CI, 0.04–0.15) or no surgery (mean difference, 0.11; 95% CI, 0.04–0.18). Baseline physical function was poorer for people who did not undergo cataract surgery than for participants who underwent first eye surgery only (mean difference in SPPB scores, 1.11 points; 95% CI, 0.17–2.05 points) or both first and second eye surgery (mean difference, 0.99 points; 95% CI, 0.07–1.91 points). The baseline characteristics of the three groups were otherwise similar (Box 2).

Box 2 Baseline (time of enrolment) characteristics of the 409 study participants*

**Table:** 

		Subsequent cataract surgery		
Parameter	All participants	First eye only	Both eyes	None
Participants	409	158	188	63
Demographic characteristics				
Age (years), mean (SD)	75.4 (5.4)	75.0 (5.8)	75.9 (5.3)	75.3 (4.7)
Sex (women)	220 (54%)	83 (53%)	99 (53%)	39 (62%)
Health status				
Comorbid conditions (FCI), mean (SD)	4.38 (2.30)	4.42 (2.43)	4.18 (2.14)	4.87 (2.32)
Medication used, mean (SD)	4.67 (3.78)	4.91 (4.51)	4.24 (3.23)	5.35 (3.18)
Five or more medications used	170 (42%)	65 (43%)	71 (39%)	35 (56%)
Antidepressants used	43 (11%)	16 (11%)	19 (11%)	8 (13%)
Vision status				
Binocular habitual visual acuity (logMAR), mean (SD)	0.27 (0.21)	0.23 (0.19)	0.32 (0.22)	0.21 (0.20)
Binocular contrast sensitivity (log units), mean (SD)	1.48 (0.21)	1.48 (0.20)	1.47 (0.22)	1.50 (0.21)
Anisometropia ≥ 1.00 dioptre	198/375 (53%)	85/141 (60%)	86/173 (50%)	27/61 (44%)
Moderate stereopsis (140 seconds of arc)	146/409 (36%)	58/158 (37%)	63/188 (34%)	25/63 (40%)
Physical function				
Weekly activity (hours), mean (SD)	44.1 (24.8)	45.4 (24.8)	44.2(24.2)	40.2 (26.5)
Physical function (SPPB score), mean (SD)	8.68 (2.59)	8.91 (2.67)	8.79 (2.50)	7.80 (2.51)
Gait speed (m/s), mean (SD)	0.95 (0.42)	0.98 (0.31)	0.94 (0.53)	0.89 (0.30)
Standing balance (seconds), mean (SD)	50.8 (12.0)	50.4 (12.3)	51.8 (11.6)	48.8 (12.0)
Sit‐to‐stand (stands per second), mean (SD)	0.37 (0.52)	0.36 (0.17)	0.40 (0.74)	0.29 (0.16)

FCI = functional comorbidity index; logMAR = logarithm of the minimum angle of resolution; SPPB = Short Physical Performance Battery; SD = standard deviation.

* Missing data (first eye surgery only/first and second eye surgery/no surgery): date of birth (0/0/2 participants), height/weight (3/1/0), total medications (7/5/1), auto‐refraction of either eye (34/0/0), bilateral contrast sensitivity (0/0/2), gait velocity (1/3/2), standing balance (4/2/1), sit‐to‐stand (7/7/2), weekly activity hours (1/0/0).

For the 118 participants who underwent first and second eye cataract surgery and participated in all follow‐ups, binocular habitual visual acuity and bilateral log contrast sensitivity improved after each operation. Mean anisometropia was 1.38 (SD, 1.71) dioptres at baseline, 2.68 (SD, 2.94) dioptres after first eye surgery, and 1.12 (SD, 1.72) dioptres after second eye surgery; 58 participants (52%) had anisometropia of 1.00 dioptre or more at baseline, 85 after the first operation (79%), and 36 after the second operation (33%). Mean refractive error was lower after surgery than at baseline (first operated eye: 1.08 [SD, 1.86] *v* 2.62 [SD, 1.23] dioptres; second operated eye: 1.31 [SD, 1.65] *v* 2.52 [SD, 1.87] dioptres) (Box 3).

Box 3 Visual characteristics before and after first eye and second eye cataract surgery for 118 participants who underwent both first and second eye surgery and participated in all follow‐ups*

**Table:** 

Parameter	Pre‐surgery	After first eye surgery	After second eye surgery	*P*		
				Pre‐surgery *v* first eye operation	Pre‐surgery *v* second eye operation	First *v* second eye operation
Binocular habitual visual acuity (logMAR), mean (SD)	0.32 (0.21)	0.15 (0.17)	0.07 (0.15)	< 0.001	< 0.001	< 0.001
First operated eye	0.51 (0.28)	0.19 (0.21)	0.14 (0.18)	< 0.001	< 0.001	0.020
Second operated eye	0.40 (0.23)	0.54 (0.27)	0.19 (0.19)	< 0.001	< 0.001	< 0.001
Binocular contrast sensitivity (log units), mean (SD)	1.48 (0.22)	1.58 (0.16)	1.64 (0.13)	< 0.001	< 0.001	< 0.001
First operated eye	1.22 (0.33)	1.48 (0.17)	1.49 (0.20)	< 0.001	< 0.001	0.78
Second operated eye	1.31 (0.26)	1.22 (0.31)	1.46 (0.20)	< 0.001	< 0.001	< 0.001
Anisometropia (dioptre), mean (SD)	1.38 (1.71)	2.68 (2.94)	1.12 (1.72)	< 0.001	0.26	< 0.001
Anisometropia ≥ 1.00 dioptre	58/112 (52%)	85/107 (79%)	36/109 (33%)	< 0.001	0.009	< 0.001
Moderate stereopsis (140 seconds of arc)	41 (35%)	34 (29%)	59 (50%)	0.28	0.020	< 0.001
First operated eye parameters						
Refractive error (vector length, dioptre), mean (SD)	2.62 (2.28)	1.08 (1.86)	1.11 (1.23)	<0.001	< 0.001	0.85
Changed spectacle refraction (≥ 0.75 dioptre)	—	46/110 (42%)	15/110 (14%)	—	—	< 0.001
Second operated eye parameters						
Refractive error (vector length; dioptre), mean (SD)	2.52 (1.87)	2.65 (2.23)	1.31 (1.65)	0.35	< 0.001	< 0.001
Changed spectacle refraction (≥ 0.75 dioptre)	—	40/113 (35%)	24/113 (21%)	—	—	0.035
Habitual distance spectacle correction						
No spectacles	48 (41%)	84 (71%)	96 (82%)	0.13	0.010	0.10
Single vision	21 (18%)	6 (5%)	4 (3%)	—	—	—
Multifocal/bifocal	47 (40%)	26 (22%)	17 (15%)	—	—	—
Contact lens	2 (2%)	2 (2%)	0 (0%)	—	—	—

logMAR = logarithm of the minimum angle of resolution; SD = standard deviation.

* General linear models to account for inherent correlation of the repeated measures. Missing data: before first operation, auto‐refraction/first eye (five), auto‐refraction/second eye (three), contrast sensitivity/first eye (one); after first operation, auto‐refraction/first eye (seven), auto‐refraction/second eye (ten), bilateral habitual visual acuity (four), habitual visual acuity/first eye (four), habitual visual acuity/second eye (three), bilateral contrast sensitivity (four), contrast sensitivity/first eye (four), contrast sensitivity/first eye (four); after second eye: auto‐refraction/first eye (eight), auto‐refraction/second eye (seven), bilateral habitual visual acuity (two), habitual visual acuity/first eye (two), habitual visual acuity/second eye (three), habitual correction (one), bilateral contrast sensitivity (two), contrast sensitivity/first eye (two), contrast sensitivity/first eye (two).

### Falls incidence

The participants returned 5139 completed falls calendars by mail (of 7828 expected; 66%); interviewers collected further falls data from 379 people in follow‐up phone calls. Age‐ and sex‐adjusted falls incidence was 1.17 (95% CI, 0.95–1.43) per year before surgery, 0.81 (95% CI, 0.63–1.04) per year after first eye surgery, and 0.41 (95% CI, 0.29–0.57) per year after second eye surgery (Box 4).

Box 4 **Falls and annual incidence of falls, by eye cataract surgery status**

**Table:** 

Parameter	Pre‐surgery	Between first and second eye surgery	After second eye surgery
Participants	409	346	188
History of falls (participants)	149 (36%)	—	—
Falls during study period (participants)	133 (32%)	109 (32%)	31 (16%)
More than two falls	62 (15%)	42 (12%)	7 (4%)
Falls in preceding year, mean number (SD)	0.88 (2.20)	—	—
Range	0–26	—	—
Falls during study period, mean number (SD)	0.80 (2.19)	0.67 (1.67)	0.21 (0.54)
Range	0–31	0–15	0–4
Observation period (days), mean (SD)	248.9 (219.8)	312.9 (218.2)	202.8 (66.4)
Range	1–761	0–753	34–485
Annual incidence, raw (95% CI)	1.17 (0.96–1.43)	0.82 (0.64–1.06)*	0.40 (0.29–0.57)
Range (falls per year)	0–19	0–25	0–7
Annual incidence, age‐ and sex‐adjusted (95% CI)	1.17 (0.95–1.43)	0.81 (0.63–1.04)	0.41 (0.29–0.57)
Incidence rate ratio (first operation *v* pre‐surgery) (95% CI)	—	0.69 (0.54–0.89)	—
Incidence rate ratio (second *v* first operation) (95% CI)	—	—	0.50 (0.33–0.76)

CI = confidence interval; IRR = incidence rate ratio; SD = standard deviation.

* Excludes one patient who had bilateral cataract surgery in one day.

In an analysis limited to the 118 participants who underwent second eye surgery and participated in all follow‐ups, the age‐ and sex‐adjusted incidence was 0.80 (95% CI, 0.55–1.15) falls per year before surgery, 0.81 (95% CI, 0.57–1.15) falls per year after first eye surgery, and 0.32 falls per year (95% CI, 0.21–0.50) after second eye surgery (Box 5).

Box 5 Age‐ and sex‐adjusted incidence of falls for 118 participants who underwent both first and second eye surgery and participated in all follow‐ups, by eye cataract surgery status*

**Figure:**
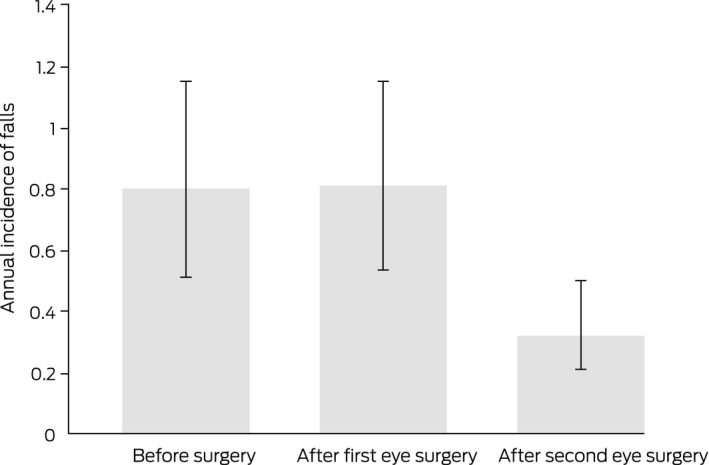
* Estimates are exponentiated marginal predictions derived from the generalised linear model, weighted by study group age and sex distribution.

## Discussion

Cataract is a major cause of vision impairment around the world,^25^ but whether cataract surgery is equitably resourced is debated in Australia^26^ and overseas.^4^ We report evidence that timely access to both first and second eye cataract surgery is important for optimising visual function and mobility in older people with bilateral cataract. In Australia, about 250 000 cataract operations are performed each year, one‐third in public hospitals.^2^ An earlier Australian analysis found that the rate of falls is particularly high among older people with bilateral cataract on public hospital waiting lists, and that half of these falls cause injuries.^21^ We found that the reduction in fall rate was greater following second eye surgery, when binocular visual function is fully restored.

Our findings clarify some apparent conflicts between earlier reports. Some authors have analysed administrative data to investigate the impact of cataract surgery on the incidence of falls leading to hospitalisation.^8^, ^9^ In a study of matched United States Medicare beneficiaries with cataract who had or had not undergone cataract surgery, surgery was associated with a lower rate of falls (adjusted odds ratio, 0.84; 95% CI, 0.81–0.87).^9^ In contrast, a Western Australian analysis found that the risk of falls increased after both first and second eye cataract surgery.^8^ The only relevant RCTs found that the effect of first eye surgery on fall risk was statistically significant,^6^ but not that of second eye cataract surgery.^7^ Our finding that second eye surgery had a greater impact on fall incidence is consistent with that of a smaller Australian cohort study (55 participants).^27^

Fewer than half the participants in our study underwent both first and second eye surgery within the 24 months examined. The profiles of participants who underwent no surgery, first eye surgery, or surgery on both eyes were similar. People with poorer vision might be expected to undergo surgery sooner, but the differences in physical function by surgical status suggests that external factors, such as competing health priorities and capabilities, may influence the timing of surgery.

As expected, measures of visual performance improved after cataract surgery, but vector analysis identified anisometropia (that is, between‐eye difference in refractive power) of at least 1.0 dioptre between operations in 79% of participants who underwent surgery on both eyes. We had previously found that spectacle prescriptions changed within three months of surgery for only 102 of 196 people who underwent first eye surgery; the 63 participants with large changes in spectacle power (0.75 dioptre or more) were at greatest risk of falls.^11^ These findings indicate that the time between first and second eye cataract surgery should be minimised to allow timely and appropriate spectacle updates after the second operation, which reduces between‐eye differences in refractive error.

### Limitations

Causal conclusions cannot be drawn from our observational study. Not all participants underwent first and second eye cataract surgery, but our analyses were adjusted for the different exposure periods. We collected comprehensive data on visual status, physical function, and health‐related risk factors for falls to characterise our population and to explore possible sources of selection bias, but some assessments were not completed because of the timing of surgery. The primary outcome, fall incidence, was based on self‐report and subject to reporting bias, but prospective monthly falls reporting is recommended for measuring this outcome.^20^ As only 25% of potential participants agreed to participate in our study, they were probably not representative of all candidates for cataract surgery or of those on public hospital waiting lists for cataract surgery, but were perhaps more mobile candidates who could travel for study assessments. We excluded residents of aged care facilities. Our participants probably came from lower socio‐economic status backgrounds than patients who undergo cataract surgery in private sector services. Many of these limitations could be averted in the randomised control trial we are planning.

## Conclusion

Our study adds to the body of evidence supporting investment in timely access to cataract surgery for older people, as it is cost‐effective for improving vision and preventing falls.^28^ Older people with cataract in Australia can wait for substantial periods for both first and second eye cataract surgery in the public hospital system. The problem has been exacerbated by deferral of elective surgery during the coronavirus disease 2019 (COVID‐19) pandemic, and particularly affects people who rely on public hospital services. Our findings indicate that timely and equitable access to cataract surgery is needed to prevent injuries and to promote healthy ageing.

## Open access

Open access publishing facilitated by University of New South Wales, as part of the Wiley ‐ University of New South Wales agreement via the Council of Australian University Librarians.

## Competing interests

No relevant disclosures.

## Supporting information


Appendix
