# Supplementary material for: The incidence of falls after first and second eye cataract surgery: a longitudinal cohort study
Source: Med J Aust. 2022 Jun 15;217(2):94–9. doi: 10.5694/mja2.51611 (PMC9546129; doi:10.5694/mja2.51611)
Supplement: Supplementary file 1 — Appendix [file MJA2-217-94-s001.pdf]

The Medical Journal of Australia

Supporting Information

Supplementary methods

This appendix was part of the submitted manuscript and has been peer reviewed.
It is posted as supplied by the authors.

Appendix to: Keay L, Ho KC, Rogers K, et al. The incidence of falls after first and second eye cataract surgery: a longitudinal cohort study. *Med J Aust* 2022; doi: 10.5694/mja2.51611.

Table. Schedule of assessments in the FOCUS study [adapted from Keay et al 2014].[1]

Recruitment phase	Initial		Phase 2	
Study visit	Baseline*	Follow-up	Baseline*	Follow-up
**Demographic characteristics**	✓	-	✓	-
**Vision status**				
Habitual correction	✓	✓	✓	✓
Refractive correction (focimeter)	✓	✓	✓	✓
Autorefraction	✓	✓	✓	✓
Distance vision	✓	✓	✓	✓
Contrast sensitivity	✓	✓	✓	✓
Visual fields	✓	-	✓	-
Stereopsis (Titmus Fly stereo test)	✓	✓	✓	✓
Ocular dominance	✓	-	✓	-
Pupil size	✓	-	✓	-
**Quality of life**				
Catquest-9SF	✓	✓	✓	✓
EQ-5D-5L	✓	✓	✓	✓
**Medical information**				
Medications and medical history	✓	✓	✓	✓
**Health service use**				
Cataract referral and surgery waiting times	✓	✓	✓	✓
**Independent mobility**				
Driving Habits Questionnaire	✓	✓	x	x
Falls (Short Falls Efficacy Scale)	✓	✓	✓	✓
Incidental and Planned Exercise Questionnaire	✓	✓	✓	✓
**Mood and socialisation**				
Keele Assessment of Participation	✓	✓	x	x
Positive and Negative Affect Scale	✓	✓	x	x
Geriatric Depression Scale	✓	✓	x	x
**Physical assessment**				
Height	✓	-	✓	-
Weight	✓	-	✓	-
Standing balance	✓	✓	✓	✓
Sit-to-stand five times	✓	✓	✓	✓
Gait speed (timed 4m walk)	✓	✓	✓	✓

(✓): performed, (-): not planned and not performed, (x): discontinued in Phase 2 of study.†

*At the completion of the baseline assessment, the monthly falls calendar is provided and the procedure for its completion explained to the participant.

† Every effort was made to support enrolment in Phase 2 of this study despite limited resources. This included modifying the protocol to remove several questionnaires on secondary outcomes in Phase 2 of data collection. Published analyses of these secondary outcomes include those of driving patterns[2] and depressive symptoms.[3] Our published analysis of the impact of first eye surgery on falls was based on the preliminary dataset of 329 patients enrolled between October 2013 and August 2015.[4]

References

1. Keay L, Palagyi A, McCluskey P, et al. Falls in Older people with Cataract, a longitudinal evalUation of impact and riSk: the FOCUS study protocol. *Inj Prev* 2014;20:e7.
2. Keay L, Palagyi A, Do V, et al. Vision and driving status of older Australians with cataract: an investigation of public hospital waiting lists. *Clin Exp Optom* 2016;99:449-455.
3. Palagyi A, Rogers K, Meuleners L, et al. Depressive symptoms in older adults awaiting cataract surgery. *Clin Exp Ophthalmol* 2016;44:789-796.
4. Palagyi A, Morlet N, McCluskey P, et al. Visual and refractive associations with falls after first-eye cataract surgery. *J Cataract Refract Surg* 2017;43:1313-1321.
